# A retrospective cohort study of 12,306 pediatric COVID-19 patients in the United States

**DOI:** 10.1038/s41598-021-89553-1

**Published:** 2021-05-13

**Authors:** Vibhu Parcha, Katherine S. Booker, Rajat Kalra, Seth Kuranz, Lorenzo Berra, Garima Arora, Pankaj Arora

**Affiliations:** 1 Division of Cardiovascular Disease, University of Alabama at Birmingham, 1670 University Boulevard, Volker Hall B140, Birmingham, AL 35294-0019 USA; 2 Department of Internal Medicine, Abbott Northwestern Hospital, Minneapolis, MN USA; 3 Division of Hospital Medicine, Children’s Minnesota, Minneapolis, MN USA; 4 Cardiovascular Division, University of Minnesota, Minneapolis, MN USA; 5 TriNetX, Inc., Cambridge, MA USA; 6 Anesthesia & Critical Care, Pulmonary Medicine, Massachusetts General Hospital, Boston, MA USA; 7 Section of Cardiology, Birmingham Veterans Affairs Medical Center, Birmingham, AL USA

**Keywords:** Diseases, Health care, Risk factors

## Abstract

Children and adolescents account for ~ 13% of total COVID-19 cases in the United States. However, little is known about the nature of the illness in children. The reopening of schools underlines the importance of understanding the epidemiology of pediatric COVID-19 infections. We sought to assess the clinical characteristics and outcomes in pediatric COVID-19 patients. We conducted a retrospective cross-sectional analysis of pediatric patients diagnosed with COVID-19 from healthcare organizations in the United States. The study outcomes (hospitalization, mechanical ventilation, critical care) were assessed using logistic regression. The subgroups of sex and race were compared after propensity score matching. Among 12,306 children with lab-confirmed COVID-19, 16.5% presented with respiratory symptoms (cough, dyspnea), 13.9% had gastrointestinal symptoms (nausea, vomiting, diarrhea, abdominal pain), 8.1% had dermatological symptoms (rash), 4.8% had neurological (headache), and 18.8% had other non-specific symptoms (fever, malaise, myalgia, arthralgia and disturbances of smell or taste). In the study cohort, the hospitalization frequency was 5.3%, with 17.6% needing critical care services and 4.1% requiring mechanical ventilation. Following propensity score matching, the risk of all outcomes was similar between males and females. Following propensity score matching, the risk of hospitalization was greater in non-Hispanic Black (RR 1.97 [95% CI 1.49–2.61]) and Hispanic children (RR 1.31 [95% CI 1.03–1.78]) compared with non-Hispanic Whites. In the pediatric population infected with COVID-19, a substantial proportion were hospitalized due to the illness and developed adverse clinical outcomes.

## Introduction

Over 4.2 million children in the United States have tested positive for coronavirus disease-2019 (COVID-19) since the onset of the pandemic^1,2^. In comparison with adults, preliminary reports suggest that children (< 18 years of age) have relatively lower odds of adverse clinical outcomes associated with COVID-19^3–8^. The lower observed prevalence of COVID-19 in the pediatric age-group worldwide is partially attributed to widespread school closures in response to the pandemic^7,9,10^. Furthermore, challenges in the adequate screening and testing of children, especially those who are asymptomatic or minimally symptomatic, may have also contributed to the underreporting of COVID-19 in children. The cautious reopening of schools in the United States and other countries has occurred in the backdrop of an increased possibility of community transmission of COVID-19 among children in schools^7,8,10^. Thus, it is important to characterize the demographic, clinical characteristics, and outcomes in children infected with COVID-19. There are limited data, especially from the United States, describing the demographics, clinical characteristics, and outcomes of lab-confirmed COVID-19 children^3–8^. We present the findings of an investigation evaluating the clinical characteristics, comorbidities, and complications in 12,306 lab-confirmed COVID-19 patients from a multicenter federated healthcare network electronic health record database.

## Results

**Figure 1:**
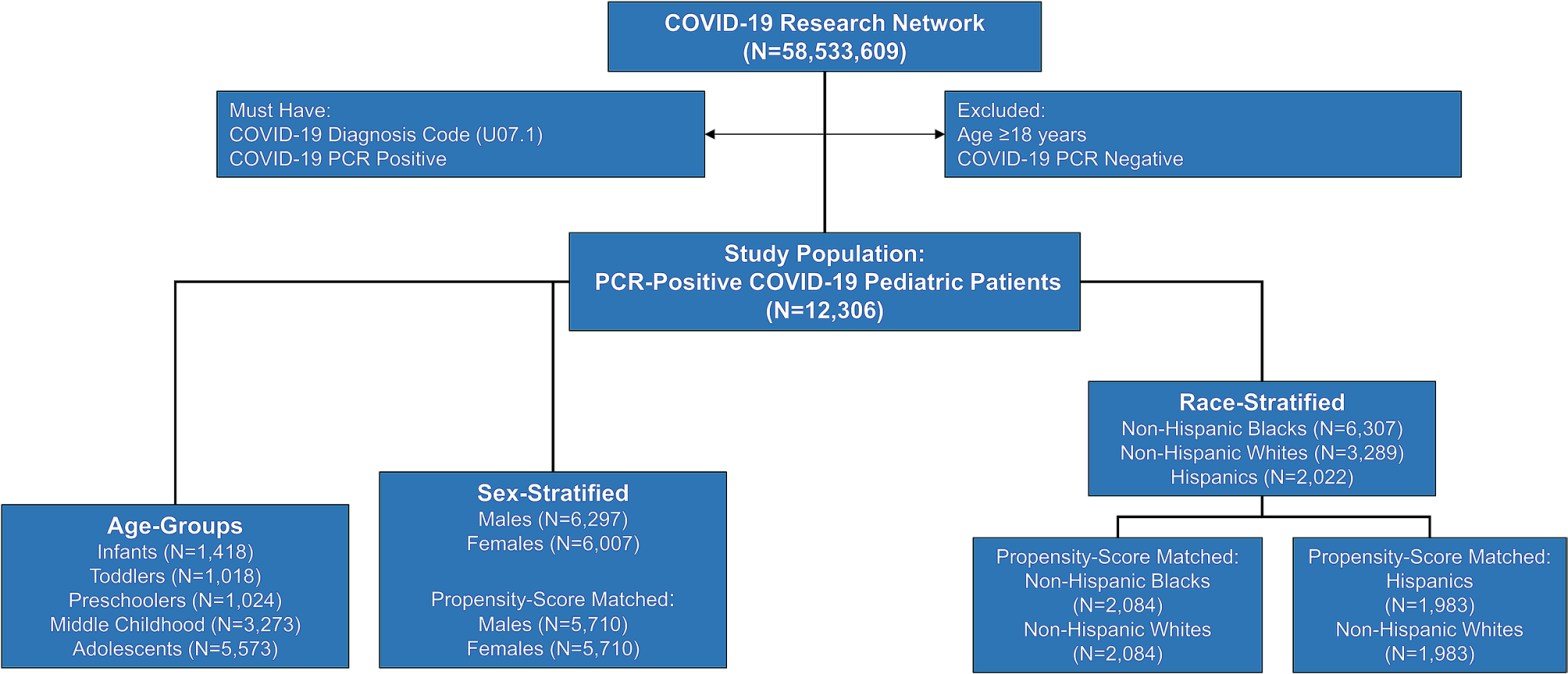
Derivation of study population.

**Figure 2:**
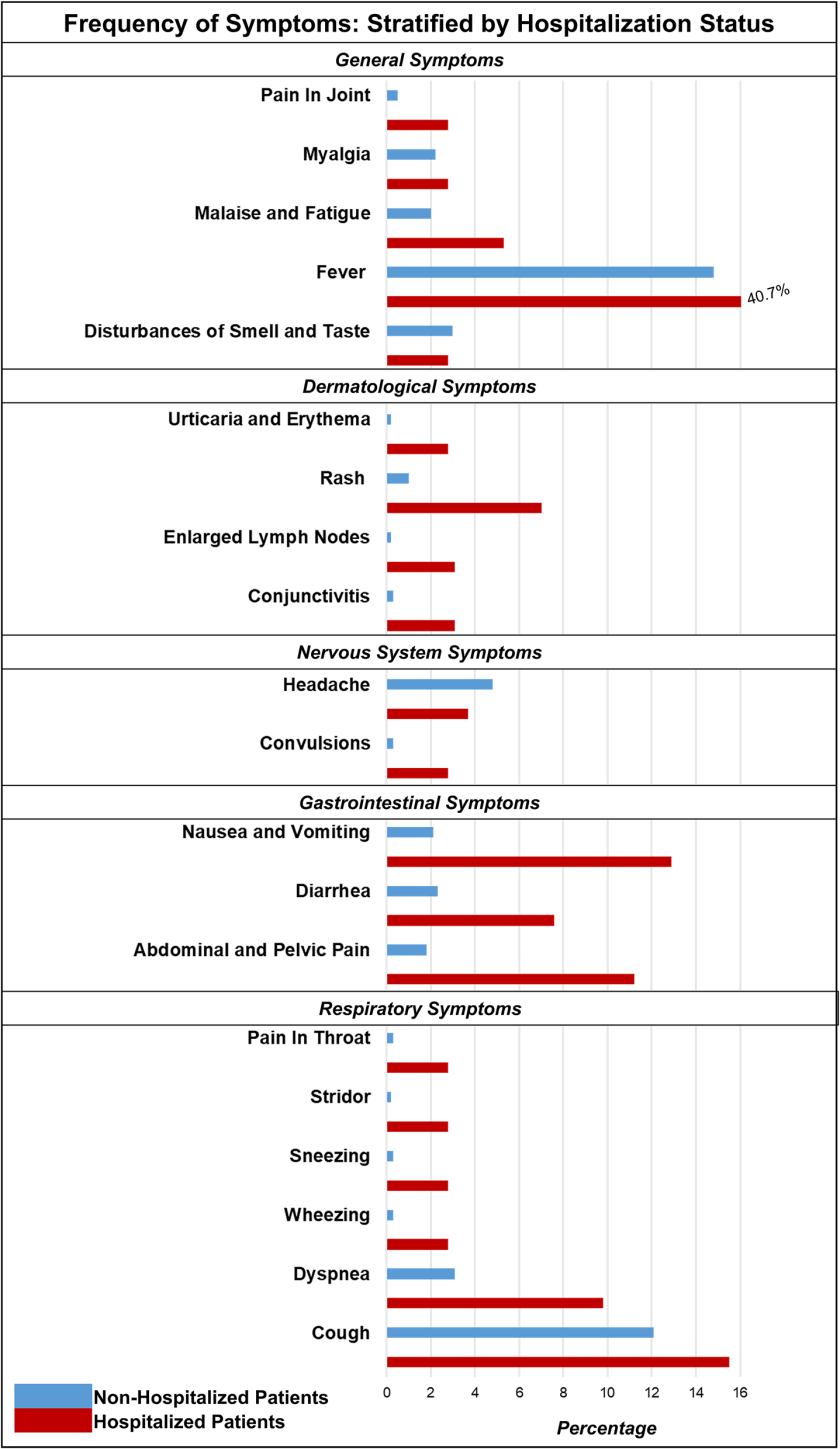
Frequency of symptoms in pediatric COVID-19 patients: stratified by hospitalization.

We identified 12,306 children with lab-confirmed COVID-19 in our cohort from 33 healthcare organizations, which are part of the TriNetX Research Network. Figure 1 describes the derivation of the study population. The geographic distribution of the cohort contributing to this analysis was composed of 43.0% patients (5289) from the Southern region, 22.9% (2814) patients from the Western region, 22.2% (2734) patients from the Midwestern region, and 7.2% (886) patients from the Northeastern region. The clinical characteristics of the overall study population stratified by hospitalization status are described in Table 1. At the time of presentation, the children who were hospitalized had a greater prevalence of fever, respiratory, gastrointestinal symptoms, and a relatively higher prevalence comorbidities. The frequency of presenting symptoms is shown in Fig. 2. Children who were hospitalized had relatively lower hemoglobin and neutrophil count. Among the study population, only 25.1% of children had at least one of the typical symptoms (fever, cough, or shortness of breath), and 9.9% of children had at least two typical symptoms. Nearly three-fourths (74.9%) of the children did not have any of the typical COVID-19 symptoms. In the study population, 16.5% presented with respiratory symptoms (cough, dyspnea), 13.9% had gastrointestinal symptoms (nausea, vomiting, diarrhea, abdominal pain), 8.1% had dermatological symptoms (rash), 4.8% had neurological (headache), and 18.8% had other non-specific symptoms (fever, malaise, myalgia, arthralgia and disturbances of smell or taste). The lab parameters of the hospitalized patients are described in Table 2. In the overall cohort, the frequency of hospitalization was 5.5%. Among those who were hospitalized (N = 672), 17.6% required critical care services (N = 118), and 4.1% required mechanical ventilation (N = 38). There were ≤ 10 deaths in the study population. Among those without any comorbidities (N = 8,297), the frequency of hospitalization was 3.5%, 0.4% required critical care, and 0.3% required mechanical ventilation.

**Table 1:** Baseline characteristics of the overall cohort.

Characteristics	Non-hospitalized cohort (total N = 11,634)		Hospitalized cohort (total N = 672)		P-value	Standardized mean difference
	Patient count	Mean ± SD or proportion	Patient count	Mean ± SD or proportion		
Age (years)	11,634	9.4 ± 5.6	672	9.0 ± 6.2	0.13	0.06
**Age groups**					< 0.001	0.53
Infant (0–1 years)	1345	11.6%	73	10.9%		
Toddlers (1–3 years)	933	8.0%	85	12.6%		
Preschoolers (3–5 years)	959	8.2%	65	9.7%		
Middle childhood (6–11 years)	3146	27.0%	127	18.9%		
Adolescents (≥ 12 years)	5256	45.1%	317	47.1%		
Male	5954	51.2%	343	51.0%	0.94	0.002
Female	5678	48.8%	329	49.0%		
**Race/ethnicity**						
Non-Hispanic Whites	6064	52.1%	243	36.2%	< 0.001	0.18
Non-Hispanic Blacks	3066	26.4%	223	33.2%	< 0.001	0.21
Hispanic	1889	16.2%	133	19.8%	< 0.001	0.16
Non-Hispanic Asians	273	2.3%	19	2.8%	0.43	0.03
Other	342	2.9%	54	8.0%	0.04	0.08
**Comorbidities**						
Cardiovascular	94	0.8%	102	15.2%	< 0.001	0.54
Gastrointestinal	334	2.9%	121	18.0%	< 0.001	0.51
Hematologic or immunologic	57	0.5%	45	6.7%	< 0.001	0.30
Malignancy	51	0.4%	34	5.1%	< 0.001	0.29
Metabolic	79	0.8%	69	10.3%	< 0.001	0.38
Neurological and Neuromuscular	252	2.2%	92	13.7%	< 0.001	0.44
Congenital or genetic defects	127	1.1%	191	28.4%	< 0.001	0.83
Renal and urologic	175	1.5%	87	13.0%	< 0.001	0.45
Respiratory	2,310	19.9%	189	28.1%	< 0.001	0.20

*SD* standard deviation.

**Table 2:** Laboratory measures of the hospitalized patients.

Characteristics	Hospitalized cohort (total N = 672)	
	Patient count	Mean ± SD
**Hematological parameters**		
Leukocytes (Cells × 10^3^/μL)	446	8.8 ± 5.0
Neutrophils (Cells × 10^3^/μL)	336	5.8 ± 3.9
Lymphocytes (per 100 leukocytes)	339	28.2 ± 9.4
Monocytes (per 100 leukocytes)	359	7.7 ± 4.6
Hemoglobin (g/dL)	462	11.9 ± 2.2
Platelets (Cells × 10^3^/µL)	446	294 ± 116
**Liver function**		
Alanine aminotransferase (IU/L)	478	42.6 ± 8.8
Albumin (g/dL)	298	3.9 ± 0.7
Alkaline phosphatase (IU/L)	309	179.0 ± 105.0
Aspartate aminotransferase (IU/L)	315	85.3 ± 57.0
Bilirubin total (mg/dL)	306	0.5 ± 0.3
Protein (g/dL)	299	6.7 ± 1.2
**Renal function**		
Creatinine (mg/dL)	384	0.7 ± 0.4
Blood urea nitrogen (mg/dL)	308	11.5 ± 5.9
**Electrolytes**		
Calcium (mg/dL)	371	9.2 ± 0.6
Potassium (mEq/L)	348	4.2 ± 0.7
Sodium (mEq/L)	379	139.0 ± 4.0
**Coagulation function**		
Activated partial thromboplastin time (s)	139	32.2 ± 10.2
Prothrombin time (s)	149	14.9 ± 6.0
INR	152	1.2 ± 0.6

*SD* standard deviation.

In our sex-stratified comparison of the study population, 5,710 pairs of male and female patients were identified after propensity-score matching. The pre and post matching propensity-score density for the subgroup comparisons are depicted in Supplementary Figs. 2–4. In the matched pairs, the risk of hospitalization was similar (RR 0.94 [95% CI 0.80–1.12]) between males (4.5%) and females (4.7%). Compared with females (0.7%), the risk ratio of requiring critical care among males (0.9%) was 0.87 (95% CI 0.55–1.12). The incidence of mechanical ventilation (RR 0.93 [95% CI 0.44–1.97]) was similar between males (0.2%) and females (0.3%).

**Figure 3:**
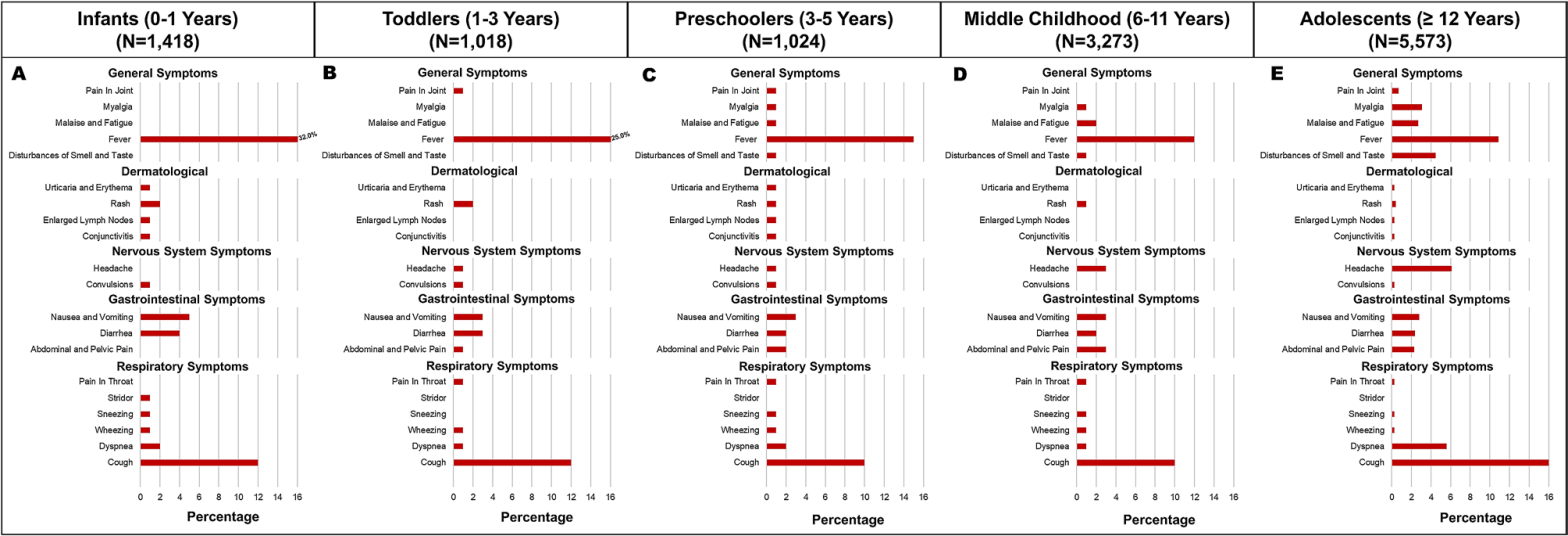
Frequency of symptoms in pediatric COVID-19 patients: stratified by age-groups.

The age-stratified presenting symptoms of the patients are described in Fig. 3. The prevalence of fever and rash was lower in adolescents compared with younger children and infants. The frequency of hospitalization, critical care requirement, and mechanical ventilation stratified by age groups is depicted in Supplementary Table 1.

After propensity-score matching, we identified 2084 pairs of non-Hispanic White and non-Hispanic Black children. The risk of hospitalization was higher in non-Hispanic Black children with COVID-19 (6.5%) compared with non-Hispanic White (3.3%) children (RR 1.97 [95% CI 1.49–2.61]). The risk of requiring critical care (RR 1.71 [95% CI 0.89–3.31]) and mechanical ventilation (RR 1.00 [95% CI 0.42–2.40]) was similar in non-Hispanic Blacks (1.2% and 0.5%) and non-Hispanic White (0.7% and 0.5%) pediatric patients. We identified 1983 propensity-score-matched pairs of Hispanic and non-Hispanic White patients. The risk of hospitalization (RR 1.31 [95% CI 1.03–1.78]) was higher in Hispanics (4.6%) compared with non-Hispanic Whites (3.5%). The risk of requiring critical care (RR 1.18 [95% CI 0.53–2.63]) and mechanical ventilation (RR 1.00 [95% CI 0.41–2.39]) was similar in Hispanic (0.7% and 0.5%) and non-Hispanic White (0.5% and 0.5%) pediatric patients. The frequency of study outcomes in non-Hispanic Asians and other racial/ethnic groups is demonstrated in Supplementary Table 2.

The descriptive trends in the case count showed a consistent increase since April 1, 2020. However, the number of hospitalization peaked in July 2020 in our cohort and was followed by a decline subsequently (Supplementary Fig. 5).

## Discussion

In this study of children with COVID-19, we observed a high prevalence of non-specific symptoms at presentation, with frequent multi-organ involvement. In our study cohort, ~ 5% were hospitalized, and among those who were hospitalized, ~ 18% required critical care, and ~ 4% needed mechanical ventilation. The clinical outcomes were similar across subgroups of sex. Non-Hispanic Black and Hispanic children with COVID-19 had a higher risk of hospitalization when compared with non-Hispanic White children. The temporal trend of the cases and hospitalization was similar to the nationwide population trends for COVID-19 cases.

We confirm the findings of prior reports from smaller populations describing the relatively milder clinical course and a relatively lower incidence of adverse clinical outcomes among children compared to adults^8,11–15^. The findings from our reports describe the wide spectrum of illness seen in children with COVID-19 across the demographic and age subgroups. The recognition of these clinical characteristics is important for the early identification and care of children with COVID-19. We observed a higher frequency of hospitalization in non-Hispanic Blacks and Hispanics. This is concordant with the recent findings reported by the Centers for Disease Control and Prevention (CDC) from the hospitals participating in the COVID-NET database^16^. The observed racial differences may be due to greater indirect viral exposure to children from racial/ethnic minorities due to the various socioeconomic impediments to implementation of infection control measures^17–24^. These racial disparities have been previously noted during the H1N1 pandemic^23,25,26^. These racial differences in COVID-19 burden are also evident in the current data from the pediatric intensive care units in the United States^27^. We observed a similar trend in COVID-19 cases and hospitalization in our database as being reported by larger tracking databases such as that by the COVKID project and the CDC’s COVID-NET^2,28,29^.

We noted that the majority of our study population were not recorded to have typical symptoms (i.e., fever, cough, or dyspnea). Prior investigations in different populations, in diverse settings, and with varying age distributions have reported that up to 40–70% of pediatric patients may present with fever and respiratory symptoms^31,32^. In contrast, some investigations have also reported a high prevalence (up to ~ 50%) of asymptomatic or mild COVID-19 infections in children^33,34^. A previous study in the United States^32^ had noted that testing indications were unclear in nearly 50% of the children, which may contribute to the low prevalence of typical symptoms at the time of diagnosis. There may also be additional factors contributing to COVID-19 testing among children, such as exposure to SARS-CoV-2 infected individuals, parental vigilance, and asymptomatic screening for travel or surgery^8,30,33–39^. However, similar to our findings, nearly all prior studies have found a relatively high proportion of non-specific signs and symptoms prompting testing among pediatric COVID-19 patients, including lethargy, malaise, myalgia, sore throat, runny nose, sneezing, gastrointestinal symptoms, and fatigue^8,30–39^. The relatively lower rates of typical symptoms noted in our study compared with other studies may also be due to the incomplete reporting of symptoms in the electronic health records, difficulty in eliciting symptomology in pre-verbal pediatric patients, relatively higher proportion of non-typical symptoms, geographic differences in the extent of spread of COVID-19^40^, and differences in the local screening and testing approaches. Further evaluation of the clinical presentation of COVID-19 among pediatric populations is needed to adequately target our screening and testing approaches.

The age-related differences in COVID-19 population prevalence^41^ and associated clinical outcomes may be a result of multiple factors^3^. Poor clinical outcomes among adult COVID-19 patients are associated with a higher comorbidity burden^42–45^. Children usually do not exhibit multiple comorbidities till a later age, and this may contribute to the lower rates of adverse clinical outcomes in children compared with adults. Pre-existing empiric immunity as a result of frequent seasonal human coronavirus infections has also been hypothesized to contribute to the lower SARS-CoV-2 infection rate among children and adolescents compared with adults^46^. In the absence of patient-level data about prior infections, the role of empiric immunity in the prevention of infection and its clinical manifestation requires further investigation. There may be other factors that may contribute to the observed lower risk in children compared with adults, such as age-dependent expression of ACE2 receptor (SARS-CoV-2 binding receptor) and androgen levels^47–49^. We also observed relatively lower neutrophil levels in the hospitalized pediatric COVID-19 patients indicating a role of age-related neutrophil recruitment in the mild manifestation of the illness in children, as reported previously^50^. The lower disease prevalence and severity in children may be due to both having lower susceptibility to COVID-19 infection and a lower likelihood of showing symptoms^3,4^.

Our findings have public health implications. The initial risk of infection transmission among children may have been limited to some extent by the early closure of schools, colleges, and universities^9,10^. The public health impact of the school closure and reopening is not completely understood^9,10^. While the presented data does not capture the potential public health impact of the various measures, these data may help to address the lacunae of clinical evidence around the patient characteristics of children with COVID-19. We observed a higher prevalence of comorbidities among those who were hospitalized following COVID-19. Identifying children who are at greater risk of complications^51–53^ may serve to create tailored strategies to screen them aggressively. Overall, our findings suggest that children and adolescents may have a milder course of illness compared with adults with COVID-19^54^. Given the high prevalence of non-specific signs and symptoms and the fact that the majority of the patients lacked typical^55^ symptoms in our investigation, increased vigilance, innovative screening, and frequent testing is required among school-going children and their immediate contacts. Routine screening tools and procedures such as daily temperature checks in school may be less effective. Our study findings may guide the resource utilization and mitigation efforts by local and federal health authorities, especially in areas with high COVID-19 incidence and prevalence. Innovative approaches, such as sentinel surveillance, random testing of children and the teachers, prioritizing children from high-risk households for COVID-19 testing, and providing education and training on the appropriate use of non-invasive pulse oximeters, may yield additional benefits and help mitigate the spread of COVID-19 among children. Implementation of these strategies may need to be enhanced among children from racial/ethnic minorities to curtail the existing COVID-19 related health disparities.

There are several limitations to our study. The patient exposure and outcomes are defined using administrative codes, which may be subject to coding errors^56,57^. Similarly, it is difficult to parse out the severity of the clinical outcomes^58–63^. Importantly, there may be significant selection bias in the children who were tested based on the indication for obtaining the tests, availability of tests, and access to testing locations and hence the eventual inclusion of the children in the study population. There were also periods early in the pandemic where testing was primarily advised for children whose clinical symptoms were thought to represent a high likelihood of COVID-19. Additionally, the information presented in our investigation is accrued from the structured data recorded in the electronic documentation. Thus, the data may be subject to inaccuracies and incomplete reporting. This may be especially important in the adequate documentation of clinical symptoms in the patient records. Furthermore, laboratory markers may not have been collected in all patients, and there may be an indication bias in the reporting of those results. Additionally, the ability to elicit symptomology is naturally limited by the nature of pediatric medicine. There may also be under-reporting of comorbidities in the administrative datasets^56,57,64–66^. We included all patients with race/ethnicity data available. While this may contribute to some degree of selection bias, it allows for accurate assessment of race-stratified outcomes. We used all-cause hospitalization as the study outcome, which makes it difficult to ascertain whether the hospitalization was due to COVID-19 or another cause. Due to lack of availability of the raw dataset, we were unable to compute the time to hospitalization and the duration of hospitalization from these data. Specific manifestations, such as the multi-systemic inflammatory syndrome^12^ in COVID-19, may not have a uniform description in electronic documentation. Thus, we could not evaluate the prevalence of this important sequela of COVID-19. Our study is also limited by the ability to detect the transmission potential of the diagnosed patients. Due to the obfuscation of counts of less than 10 for privacy concerns, we are unable to report the exact number of deaths in the overall population and subgroups.

In summary, children infected with COVID-19 present with a broad spectrum of non-specific symptoms across the age groups. Children with COVID-19 can develop severe illness requiring hospitalization and critical care, but the rates of severe illness and death are relatively low.

## Methods

### Data source

The TriNetX (Cambridge, MA) COVID-19 Research Network database was used for this study^58–63^. This research network database is a federated health research network database that incorporates and integrates the electronic health records from the participating healthcare organizations, which includes nearly 59 million patients. The research network database integrates cloud-based HIPAA-compliant real-time aggregate patient-level data from the electronic health records, which includes diagnoses, procedures, medication use, and clinical laboratory values from the contributing organizations^58–63^. The participating organizations contribute data from inpatient, outpatient, and specialty services. The TriNetX database integrates data from all the participating organizations after clearance through local data warehouses and research data repositories. To ensure patient privacy, the stored and transmitted data are de-identified at the patient, and organization level. Structured data recorded in the electronic health records are assimilated into the database after mapping the data to standard and controlled clinical terms. A rigorous data quality assessment is done to exclude records that do not meet quality standards and basic formatting requirements for adequate data representation^63^. The referential integrity is maintained to ensure comparison of data across several databases. Moreover, TriNetX software also ensures data validity by regularly monitoring the temporal trend of data volume^58–61,63^. Further details about the database are provided in the Supplementary Methods^63^. This study was deemed to be non-human subjects research by the University of Alabama at Birmingham Institutional Reviewer Board.

### Standardized coding of the database

The TriNetX database captures diagnoses using the International Statistical Classification of Diseases and Related Health Problems Clinical Modifications 10th Edition (ICD-10-CM). The database records the procedures using standardized Current Procedural Terminology (CPT) codes. The clinical lab parameters are captured using the standard Logical Observation Identifiers Names and Codes (LOINC). The individual codes chosen for defining the COVID-19 patients and for the definition of study outcomes are described in Supplementary Table 4^58–61^.

### Study population

The COVID-19 research network database was queried to identify individuals < 18 years of age and diagnosed with lab-confirmed COVID-19 between April 1, 2020 to October 31, 2020. Patients with the relevant International Statistical Classification of Diseases and Related Health Problems (ICD) codes (U07.1)^67^ or positive lab result (LOINC Codes: 94309-2, 94315-9, 94316-7, 94500-6, 94533-7, 94534-5, 94502-2, 94599-2, 9088, 40458-1) were included in the study based on the World Health Organization and Centers for Disease Control and Prevention Guidelines. We included only individuals with lab-confirmed (using positive polymerase-chain-reaction) COVID-19 results to ensure that we do not include children with suspected COVID-19, i.e., patients under investigation. We further stratified the population on the basis of age (infants [0–1 year], toddlers [1–3 years], preschoolers [3–5 years], middle childhood [6–11 years], and adolescents [12–17 years]), and sex (males and females). The population was also stratified on the basis of race/ethnicity (non-Hispanic White, non-Hispanic Black, Hispanic, non-Hispanic Asian, Others). The study population was further stratified patients based on the month of diagnosis (April, May, June, July, August, September, October) to identify the temporal trend in the cases and outcomes.

### Measures and outcomes

We identified the baseline patient characteristics, including past medical history, presenting symptoms, medications, and lab parameters. We identified the clinical features and laboratory values for lab parameters, which were identified from within the last 1 month up to the index event in the hospitalized cohort. Typical symptoms were defined as having any of the three symptoms: fever, cough, and shortness of breath, as defined by the CDC^55^. The lab-confirmed diagnosis of COVID-19 was defined as the index event. The standardized ICD-10 diagnosis codes were used to identify the history of existing medical conditions. The main study outcome was the frequency of all-cause hospitalization within 30-days of testing positive in children with COVID-19 and in the abovementioned sub-groups. The additional study outcomes included mechanical ventilation and the requirement for critical care. The administrative diagnosis and procedural codes were used for the identification of the aforementioned study outcomes (Supplementary Tables 4–6).

### Statistical analyses

We summarized the baseline characteristics as mean ± standard deviation for continuous data and as numbers and percentages for categorical data. The baseline characteristics were compared using descriptive statistics with the continuous data were compared using independent sample t-test, and categorical data were compared using the z-score. The study outcomes were compared in age and sex subgroups. All primary and secondary outcomes were reported in the overall populations and the sub-groups of age, sex, and race/ethnicity. Logistic regression was applied to obtain a propensity score for each patient using logistic regression implemented by the function LogisticRegression of the scikit-learn package in Python version 3.7^63,68^. The output was verified by repeating the propensity scoring in R version 3.4.4. Subsequently, a 1:1 matching was done using greedy nearest neighbor matching with a caliper of 0.1 pooled standard deviation^63,69^. The propensity score-matched populations were matched for age, sex, race, and comorbidities (cardiovascular, respiratory, gastrointestinal, malignancy, metabolic, hematological or immunological, neurological and neuromuscular, congenital or genetic defects, renal or urological) (Supplementary Table 6)^70^. For the protection of inadvertent disclosure of protected health information, patient counts for demographics, clinical characteristics, and outcomes if less than is reported as ≤ 10. We report the comparative risk of the study outcomes as risk ratios with 95% confidence intervals. The two-tailed type I error of 0.05 was deemed to be statistically significant. The cloud-based TriNetX analytics platform, which utilizes a combination of JAVA, R, and Python, was used for all analyses^58–61,63^.

## Supplementary Information

Supplementary Information.

## Data Availability

The data from the TriNetX COVID-19 Research database is available to member healthcare organizations through the online cloud-based TriNetX research platform available at https://www.trinetx.com/. The aggregate patient-level is integrated from the electronic health records of the member healthcare organizations, with data available for download by request at the participating institutions.
