# Supplementary material for: A retrospective cohort study of 12,306 pediatric COVID-19 patients in the United States
Source: Sci Rep. 2021 May 13;11:10231. doi: 10.1038/s41598-021-89553-1 (PMC8119690; doi:10.1038/s41598-021-89553-1)
Supplement: Supplementary file 1 — Supplementary Information. [file 41598_2021_89553_MOESM1_ESM.docx]

**A Retrospective Cohort Study of 12,306 Pediatric COVID-19 Patients in the United States**

**­­­­­­­­­­­­­­­­­­­­­­­­­**Vibhu Parcha, MD^1^, Katherine S. Booker, MD^2,3^, Rajat Kalra, MBChB, MS^4^, Seth Kuranz, PhD^5^, Lorenzo Berra, MD^6^, Garima Arora, MD^1^, Pankaj Arora, MD^1,7^

**Affiliations:**

1. Division of Cardiovascular Disease, University of Alabama at Birmingham, Birmingham, AL, USA.
2. Department of Internal Medicine, Abbott Northwestern Hospital, Minneapolis, MN, USA.
3. Division of Hospital Medicine, Children’s Minnesota, Minneapolis, MN, USA.
4. Cardiovascular Division, University of Minnesota, Minneapolis, MN, USA.
5. TriNetX, Inc., Cambridge, MA, USA.
6. Anesthesia & Critical Care, Pulmonary Medicine, Massachusetts General Hospital, Boston, MA, USA.
7. Section of Cardiology, Birmingham Veterans Affairs Medical Center, Birmingham, AL, USA.

**Supplementary Methods**

The TriNetX Research Network database is a federated health research database that integrates the electronic health records of nearly 59 million patients. The diagnoses, procedures, vital signs, and lab results are linked to a unique de-identified patient ID. The de-identified patient ID is linked with a unique de-identified encounter ID. The variables available in the dataset are present as categorical (for diagnoses, procedures, and medication) or continuous (lab results and vital signs).

The data in TriNetX datasets are:

• Primarily from healthcare care organization (HCO)’s electronic medical record (EMR) systems
• Collected for the primary purpose of providing care to patients

The data in TriNetX datasets are not:

• Claims data, data primarily collected for the purposes of billing
• Data collected for the purposes of randomized clinical trials

The majority of the HCOs are large academic medical institutions with both inpatient and outpatient facilities. Most of these HCOs are adult acute-care hospitals with multiple facilities and locations. All HCOs are currently located within the United States. The individual HCOs provide TriNetX with both inpatient and outpatient data. The data they provide is representative of the entire patient population at the HCO. Thus, the data from each HCO represents a combination of data from hospitals, urgent care centers, and primary care clinics. Most HCOs provide an average of seven years of historical data.

TriNetX typically receives data from HCOs and other data providers in one of two ways:

1. TriNetX obtains data directly from an HCO’s research repository (e.g., i2b2) into the TriNetX environment.

2. A HCO or data provider sends TriNetX data extracts in the form of CSV files.

TriNetX maps the data to a standard and controlled set of clinical terminologies. The data is then transformed into a proprietary data schema. This transformation process includes an extensive data quality assessment that includes ‘data cleaning’ that rejects records that do not meet the TriNetX quality standards of having the EMR data in the proper format to be accepted as a valid patient record.


**Supplementary Figure 1: Data Tables Relationship for TriNetX Research Network Database**

The database currently contributes to the National Institutes of Health-National Center for Advancing Translational Sciences’ National COVID Cohort Collaborative^1^, which aims to combine the electronic health records of all United States healthcare organizations. The TriNetX database has also partnered with the Food & Drug Administration’s Office of Surveillance and Epidemiology (OSE)^2^ and the Sentinel Operations Center (SOC) led by the Harvard Pilgrim Health Care Institute (HPHCI),^3^ to provide real-world surveillance data for epidemiological research. The work from this database has been cited in the Centers for Disease Control and Prevention policy guidelines^4^ regarding COVID-19 in children. The database has been used for several high-impact publications^5-9^.

There are 41 HCOs, including the University of Alabama at Birmingham, all of which are in the United States, which contributed to the data in this study. Due to contractual data-use agreement limitations and to ensure patient privacy, the TriNetX Research Network cannot share the details of the individual participant healthcare organizations. **Supplementary Table 1** table below enlists the healthcare organizations that publicly acknowledge the partnership with TriNetX Research Network.

**Supplementary Table 1. List of Organizations With Publicly Acknowledged Partnership with TriNetX** **Research Network**

| **Serial No.** | **Organization Name** | **Internet Hyperlink** |
| --- | --- | --- |
| 1 | Nationwide Children’s Hospital | <https://trinetx.com/nationwide-childrens-hospital-joins-the-trinetx-health-research-network/> |
| 2 | Children’s National Hospital | <https://www.prnewswire.com/news-releases/childrens-national-research-institute-expanding-research-resources-for-investigators-driving-multi-site-pediatric-collaboration-with-trinetx-300942916.html> |
| 3 | Boston Children’s Hospital | <https://www.prnewswire.com/news-releases/boston-childrens-hospital-accelerates-data-driven-approach-to-clinical-research-by-joining-the-trinetx-global-health-research-network-300601082.html> |
| 4 | Children’s Hospital, Colorado | <https://trinetx.com/clients/childrens-hospital-colorado/> |
| 5 | University of Alabama at Birmingham | <https://www.uab.edu/ccts/partnerships/collaborative-platforms/trinetx> |
| 6 | University of Texas Southwestern Medical Center | <https://www.utsouthwestern.edu/research/translational-medicine/doing-research/biomedical-informatics/trinetx.html> |
| 7 | John Hopkins University | <https://ictr.johnshopkins.edu/programs_resources/programs-resources/i2c/trinetx/> |
| 8 | Icahn School of Medicine at Mount Sinai | <http://researchroadmap.mssm.edu/reference/systems/trinetx/> |
| 9 | Tufts Medical Center | <https://www.tuftsctsi.org/research-services/informatics/> |
| 10 | Weil Cornell University | <https://its.weill.cornell.edu/services/research-informatics/trinetx> |
| 11 | Thomas Jefferson University | <https://www.prnewswire.com/news-releases/jefferson-joins-the-trinetx-network-to-revolutionize-clinical-trial-design-to-enhance-and-accelerate-the-development-of-new-drugs-300266109.html> |
| 12 | University of Cincinnati | <https://www.cctst.org/programs/biomedical-informatics/trinetx> |
| 13 | Ochsner Health System | <https://www.prnewswire.com/news-releases/ochsner-health-system-partners-with-trinetx-an-international-research-data-network-300270971.html> |
| 14 | Penn State College of Medicine | <https://ctsi.psu.edu/research-support/trinetx/> |
| 15 | University of Iowa | <https://icts.uiowa.edu/investigators/biomedical-informatics-core/trinetx> |
| 16 | University of Rochester Medical Center | <https://www.urmc.rochester.edu/clinical-translational-science-institute/informatics/cohort-discovery/trinetx.aspx> |
| 17 | Houston Methodist Hospital | <https://www.prnewswire.com/news-releases/houston-methodist-hospital-joins-trinetx-to-enhance-study-feasibility-academic-collaboration-and-participation-in-clinical-trials-300803126.html> |
| 18 | University of Kentucky | <https://med.uky.edu/news/university-kentucky-joins-trinetx-network> |
| 19 | University Hospital, Cleveland | <https://www.uhhospitals.org/uh-research/for-researchers/research-and-clinical-trials/for-researchers/research-toolbox/trinetx> |
| 20 | University of South Florida | <https://hscweb3.hsc.usf.edu/blog/2016/08/05/welcome-to-i2b2-our-powerful-new-research-tool/> |
| 21 | Virginia Commonwealth University | <https://cctr.vcu.edu/support/informatics/cohort-discovery/> |
| 22 | University of Tennessee Health Sciences Center | <https://tnctsi.uthsc.edu/consultation-and-services/biomedical-informatics/> |
| 22 | Medical University of South Carolina | <https://research.musc.edu/resources/sctr/spotlight/2020/06/trinetx> |
| 23 | Carilion Clinic | <https://trinetx.com/carilion-clinic-joins-trinetx/> |

**Supplementary Table 2. Clinical Outcomes Stratified by Age**

| **Outcomes** | **Infant (0-1 Years)**  **(N=1,418)** | **Toddlers (1-3 Years)**  **(N=1,018)** | **Preschoolers (3-5 Years)**  **(N=1,024)** | **Middle Childhood**  **(6-11 Years) (N=3,273)** | **Adolescents (≥ 12 Years)**  **(N=5,573)** |
| --- | --- | --- | --- | --- | --- |
| Hospitalization | 5.1% | 8.4% | 6.4% | 3.9% | 5.7% |
| Critical Care Requirement | 1.2% | 1.0% | 1.0% | 0.9% | 1.0% |
| Mechanical Ventilation | 0.7% | 1.0% | 1.0% | 0.3% | 0.3% |

**Supplementary Table 3. Clinical Outcomes in Non-Hispanic Asians and Other Racial/Ethnic Group**

| **Outcomes** | **Non-Hispanic Asians**  **(N=292)** | **Others**  **(N=342)** |
| --- | --- | --- |
| Hospitalization | 6.5% | 13.6% |
| Critical Care Requirement | 3.7% | 4.4% |
| Mechanical Ventilation | 0% | 2.1% |

**Supplementary Table 4. Diagnosis Codes Used in the Study Analyses**

The individual codes chosen for defining the COVID-19 patients and for the definition of study outcomes are described below:

| **Characteristic** | **CPT/ ICD/ LOINC Codes** |
| --- | --- |
| COVID-19 Diagnosis | U07.1 (ICD-10 COVID-19) –Lab Confirmed COVID-19  94309-2 (LOINC: SARS-CoV-2 (COVID-19) RNA [Presence] in Unspecified specimen by NAA with probe detection)[Positive]  94315-9 (SARS-related coronavirus E gene [Presence] in Unspecified specimen by NAA with probe detection)[Positive]  94316-7 (SARS-CoV-2 (COVID-19) N gene [Presence] in Unspecified specimen by NAA with probe detection)[Positive]  94500-6 (SARS-CoV-2 (COVID-19) RNA [Presence] in Respiratory specimen by NAA with probe detection)[Positive]  94533-7 (SARS-CoV-2 (COVID-19) N gene [Presence] in Respiratory specimen by NAA with probe detection)[Positive]  94534-5 (SARS-CoV-2 (COVID-19) RdRp gene [Presence] in Respiratory specimen by NAA with probe detection)[Positive]  94502-2 (SARS-related coronavirus RNA [Presence] in Respiratory specimen by NAA with probe detection)[Positive]  94559-2 (SARS-CoV-2 (COVID-19) ORF1ab region [Presence] in Respiratory specimen by NAA with probe detection)[Positive]  41458-1 (SARS coronavirus RNA [Presence] in Unspecified specimen by NAA with probe detection)[Positive] |
| **Outcomes** | |
| Mortality | “Deceased” (Known Deceased Documented) |
| Critical Care | 1013729 (CPT: Critical Care Services)  CPT: Infant or Pediatric Critical Care Services |
| Hospitalization | 1013659 (CPT: Hospital Inpatient Services)  1013699 (CPT: Initial Inpatient Consultation)  1013729 (CPT: Critical Care Services)  “Visit: Inpatient Acute”  “Visit: Inpatient Encounter”  “Visit: Inpatient Non-acute”  “Visit: Short Stay” |
| Mechanical Ventilation | 31500 (CPT: Intubation, endotracheal, emergency procedure)  1015098 (CPT: Ventilator management)  5A1935Z (ICD10: Respiratory Ventilation, Less than 24 Consecutive hours)  5A1945Z (ICD10: Respiratory Ventilation, 24-96 Consecutive hours)  5A1955Z (ICD10: Respiratory Ventilation, Greater than 96 Consecutive hours)  0BH17EZ (ICD10: Insertion of Endotracheal Airway into Trachea, Via Natural or Artificial Opening)  0BH18EZ (ICD10: Insertion of Endotracheal Airway into Trachea, Via Natural or Artificial Opening Endoscopic)  0BH13EZ (ICD10: Insertion of Endotracheal Airway into Trachea, Percutaneous Approach)  1022227 (CPT: Extracorporeal membrane oxygenation (ECMO)/ extracorporeal life support (ECLS) provided by physician)  39.65 (ICD9: Extracorporeal membrane oxygenation [ECMO]) |

**Supplementary Table 5. Diagnosis Codes for Clinical Symptoms**

| **Symptom** | **ICD-10 Code** |
| --- | --- |
| Cough | R05 |
| Dyspnea | R06.0 |
| Wheezing | R06.2 |
| Pain in Throat | R07.0 |
| Nausea and Vomiting | R11 |
| Diarrhea | R19.7 |
| Abdominal and Pelvic Pain | R10 |
| Convulsions | R56 |
| Headache | R51 |
| Urticaria and Erythema | L49-L54 |
| Rash | R21 |
| Enlarged Lymp Nodes | R59 |
| Conjunctivitis | H10 |
| Pain in Joint | M25.5 |
| Myalgia | M79.1 |
| Malaise and Fatigue | R53 |
| Fever | R50 |
| Disturbance of Smell and Taste | R43 |

**Supplementary Table 6. ICD-10 Codes for Pediatric Comorbidities**

| **Comorbidity** | **ICD-10 Code** |
| --- | --- |
| Cardiovascular | Q20, Q21.2-Q24, Q25.1-Q26, Q28.2, Q28.3, Q28.9, 02170ZP, 02170ZQ, 02170ZR, 02BK0ZZ, 02LR0ZT, 02LS0ZZ, 02LT0ZZ, 02NH0ZZ, 02RK0JZ, 02RL0JZ, 02RM0JZ, 02RP0JZ, 02RQ07Z, 02RQ0JZ, 02RR07Z, 02RR0JZ, 02SP0ZZ, 02SW0ZZ, 02U70JZ, 02UA0JZ, 02UA3JZ, 02UA4JZ, 02VR0ZT, 02WA0JZ  I34.0, I34.8, I36.0, I36.8, I37.0, I37.8  I42, I43, I51.5  I44, I45, I47, I48, I49.0  I49.1-I49.5, I49.8, I49.9, R00.1  I27.0, I27.1, I27.2, I27.81, I27.89, I27.9, I50.9, I51.7, I51.81, I63.139, I63.239, Z95.1  T82.519A, T82.529A, T82.539A, T82.599A, T82.110A, T82.111A, T82.120A, T82.121A, T82.190A, T82.191A, T82.01XA, T82.02XA, T82.03XA, T82.09XA, T82.211A, T82.212A, T82.213A, T82.218A, T82.221A, T82.222A, T82.223A, T82.228A, T82.518A, T82.528A, T82.538A, T82.598A, T82.6XXA, T82.7XXA, Z95.0, Z95.2, Z95.3, Z95.810-Z95.812, Z95.818, Z45.010, Z45.018, Z45.02, Z45.09, Z95.9, 02H40JZ, 02H40KZ, 02H43JZ, 02H44JZ, 02H44KZ, 02H60JZ, 02H60KZ, 02H63JZ, 02H63KZ, 02H63MZ, 02H64JZ, 02H64KZ, 02H70KZ, 02H73JZ, 02H73KZ, 02H73MZ, 02H74KZ, 02HA0QZ, 02HA0RS, 02HA0RZ, 02HA3QZ, 02HA3RS, 02HA4QZ, 02HA4RS, 02HK0JZ, 02HK0KZ, 02HK3JZ, 02HK3KZ, 02HK3MZ, 02HK4JZ, 02HK4KZ, 02HL0JZ, 02HL0KZ, 02HL0MZ, 02HL3JZ, 02HL3KZ, 02HL3MZ, 02HL4JZ, 02HL4KZ, 02HL4MZ, 02HN0JZ, 02HN0KZ, 02HN0MZ, 02HN3JZ, 02HN3KZ, 02HN3MZ, 02HN4JZ, 02HN4KZ, 02HN4MZ, 02WA0QZ, 02WA0RZ, 02WA3QZ, 02WA3RZ, 02WA4QZ, 02WA4RZ, 03HK0MZ, 03HK3MZ, 03HK4MZ, 03HL0MZ, 03HL3MZ, 03HL4MZ, 03WY0MZ, 03WY3MZ, 03WY4MZ, 0JH600Z, 0JH605Z, 0JH606Z, 0JH607Z, 0JH608Z, 0JH609Z, 0JH60AZ, 0JH60MZ, 0JH60PZ, 0JH630Z, 0JH635Z, 0JH636Z, 0JH637Z, 0JH638Z, 0JH639Z, 0JH63AZ, 0JH63MZ, 0JH63PZ, 0JH70MZ, 0JH73MZ, 0JH800Z, 0JH805Z, 0JH806Z, 0JH807Z, 0JH808Z, 0JH809Z, 0JH80AZ, 0JH80MZ, 0JH80PZ, 0JH830Z, 0JH835Z, 0JH836Z, 0JH837Z, 0JH838Z, 0JH839Z, 0JH83AZ, 0JH83MZ, 0JH83PZ, 0JWT0MZ, 0JWT0PZ, 0JWT3MZ, 0JWT3PZ, 0JWTXMZ, 4B02XSZ, 4B02XTZ, 5A02110, 5A02116, 5A0211D, 5A02210, 5A02216, 5A0221D  T86.20-T86.22, Z94.1, 02YA0Z0, 02YA0Z1, 02YA0Z2 |
| Gastrointestinal | Q39.0-Q39.4, Q41-Q45  K73, K74, K75.4, K760-K763, K765, K768  K50, K51  I82.0, K55.1, K56.2, K59.3, Z98.0, Z90.3, Z90.49, 0CT70ZZ, 0CT7XZZ, 0D13079, 0D1307A, 0D1307B, 0D1607A, 0D160ZA, 0DT50ZZ, 0DT54ZZ, 0DT57ZZ, 0DT58ZZ, 0DT60ZZ, 0DT64ZZ, 0DT67ZZ, 0DT68ZZ, 0DT80ZZ, 0DT84ZZ, 0DT87ZZ, 0DT88ZZ, 0DT90ZZ, 0DT94ZZ, 0DT97ZZ, 0DT98ZZ, 0DTE0ZZ, 0DTE4ZZ, 0DTE7ZZ, 0DTE8ZZ, 0FT00ZZ, 0FT04ZZ, 0FTG0ZZ, 0FTG4ZZ  K94.20, K94.22, K94.23, K94.29, Z93.1-Z93.4, Z43.1-Z43.4, Z46.51, Z46.59, 0D11074, 0D110J4, 0D110K4, 0D110Z4, 0D113J4, 0D11474, 0D114J4, 0D114K4, 0D114Z4, 0D15074, 0D150J4, 0D150K4, 0D150Z4, 0D153J4, 0D15474, 0D154J4, 0D154K4, 0D154Z4, 0D16074, 0D160J4, 0D160J9, 0D160JA, 0D160K4, 0D160K9, 0D160KA, 0D160Z4, 0D163J4, 0D16474, 0D164J4, 0D164J9, 0D164JA, 0D164K4, 0D164K9, 0D164KA, 0D164Z4, 0D16874, 0D168J4, 0D168J9, 0D168JA, 0D168K4, 0D168K9, 0D168KA, 0D168Z4, 0D1B0Z4, 0D1B4Z4, 0D1B8Z4, 0D1H0Z4, 0D1H4Z4, 0D1H8Z4, 0D1K0Z4, 0D1K4Z4, 0D1K8Z4, 0D1L0Z4, 0D1L4Z4, 0D1L8Z4, 0D1N0Z4, 0D1N4Z4, 0D1N8Z4, 0D20X0Z, 0D20XUZ, 0D20XYZ, 0D787ZZ, 0D7E7ZZ, 0DBB7ZZ, 0DH50DZ, 0DH50UZ, 0DH53DZ, 0DH53UZ, 0DH54DZ, 0DH54UZ, 0DH57DZ, 0DH57UZ, 0DH58DZ, 0DH58UZ, 0DH63UZ, 0DH64UZ, 0DHA3UZ, 0DHA4UZ, 0DHA8UZ, 0DN87ZZ, 0DNE7ZZ, 0DW04UZ, 0DW08UZ, 0WQFXZ2, 3E1G78Z, 3E1H78Z  T86.40-T86.42, T86.890, T86.891, T86.899, T86.850, T86.851, T86.859, Z94.4, Z94.82, Z94.83, 0DY80Z0, 0DY80Z1, 0DY80Z2, 0DYE0Z0, 0DYE0Z1, 0DYE0Z2, 0FY00Z0, 0FY00Z1, 0FY00Z2, 0FYG0Z0, 0FYG0Z1, 0FYG0Z2, 3E030U0, 3E030U1, 3E033U0, 3E033U1, 3E0J3U0, 3E0J3U1, 3E0J7U0, 3E0J7U1, 3E0J8U0, 3E0J8U1 |
| Hematologic or Immunologic | D55-D58  D60-D61, D71  D80-D89, D72.0, M30.3, M35.9  D66, D68.2, D69.41, D69.42, D69.49  D70.0, D70.4  D76.1-D76.3  D86.9  B20-B24  M30.0, M31.0, M31.1, M31.30, M31.4, M31.6  M32.10, M33.90, M34.0, M34.1, M34.9  07TP0ZZ, 07TP4ZZ  07YP0Z0, 07YP0Z1, 07YP0Z2, 30230AZ, 30230G0, 30230G1, 30230X0, 30230X1, 30230Y0, 30230Y1, 30233AZ, 30233G0, 30233G1, 30233X0, 30233X1, 30233Y0, 30233Y1, 30240AZ, 30240G0, 30240G1, 30240X0, 30240X1, 30240Y0, 30240Y1, 30243AZ, 30243G0, 30243G1, 30243X0, 30243X1, 30243Y0, 30243Y1, 30250G0, 30250G1, 30250X0, 30250X1, 30250Y0, 30250Y1, 30253G0, 30253G1, 30253X0, 30253X1, 30253Y0, 30253Y1, 30260G0, 30260G1, 30260X0, 30260X1, 30260Y0, 30260Y1, 30263G0, 30263G1, 30263X0, 30263X1, 30263Y0, 30263Y1 |
| Malignancy | C00-C96, D01-D09, D3A.0, D37-D49, Q85.0, 3E00X05, 3E01305, 3E02305, 3E03005, 3E03305, 3E04005, 3E04305, 3E05005, 3E05305, 3E06005, 3E06305, 3E0A305, 3E0F305, 3E0F705, 3E0F805, 3E0G305, 3E0G705, 3E0G805, 3E0H305, 3E0H705, 3E0H805, 3E0J305, 3E0J705, 3E0J805, 3E0K305, 3E0K705, 3E0K805, 3E0L305, 3E0L705, 3E0M305, 3E0M705, 3E0N305, 3E0N705, 3E0N805, 3E0P305, 3E0P705, 3E0P805, 3E0Q305, 3E0Q705, 3E0R305, 3E0S305, 3E0V305, 3E0W305, 3E0Y305, 3E0Y705  T86.00-T86.02, T86.09, Z94.81, Z94.84 |
| Metabolic | E70.0, E70.2, E70.3, E70.4, E70.8, E71.0-E71.5, E72.0-E72.4, E72.8, E72.9  E74.0-E74.4, E74.8, E74.9  E75, E77.0, E77.1, E78.0-E78.4, E78.5-E78.9, E88.1, E88.8  E76.0-E76.3, E85  277.4, E79.1, E79.8, E80.4-E80.7, E83.0, E83.1, E83.3, E83.4, D84.1, E88, H49.8  E00.9, E23.0, E23.2, E22.2, E23.3, E23.7, E24.0, E24.2, E24.3, E24.8, E24.9, E26.81, E25.0, E25.8, E25.9, 0GT00ZZ, 0GT04ZZ, 0GT40ZZ, 0GT44ZZ, 0GTK0ZZ, 0GTK4ZZ, 0GTR0ZZ, 0GTR4ZZ, 0UT20ZZ, 0UT24ZZ, 0UT27ZZ, 0UT28ZZ, 0UT2FZZ, 0UT40ZZ, 0UT44ZZ, 0UT47ZZ, 0UT48ZZ, 0UT70ZZ, 0UT74ZZ, 0UT90ZZ, 0UT94ZZ, 0UT97ZZ, 0UT98ZZ, 0UT9FZZ, 0UTC0ZZ, 0UTC7ZZ, 0UTC8ZZ, 0VTC0ZZ, 0VTC4ZZ, 0W4M070, 0W4M0J0, 0W4M0K0, 0W4M0Z0, 0W4N071, 0W4N0J1, 0W4N0K1, 0W4N0Z1  Z46.81, Z96.41, 0JH60VZ, 0JH63VZ, 0JH70VZ, 0JH73VZ, 0JH80VZ, 0JH83VZ, 0JHD0VZ, 0JHD3VZ, 0JHF0VZ, 0JHF3VZ, 0JHG0VZ, 0JHG3VZ, 0JHH0VZ, 0JHH3VZ, 0JHL0VZ, 0JHL3VZ, 0JHM0VZ, 0JHM3VZ, 0JHN0VZ, 0JHN3VZ, 0JHP0VZ, 0JHP3VZ, 0JHT0VZ, 0JHT3VZ |
| Neurological and Neuromuscular | Q00-Q07, G90.1  F71-F73  E75.0, E75.1, E75.2, E75.4, F84.2, G11.1-G11.4, G11.8, G11.9, G12.0- G12.2, G12.8, G12.9, G31.01, G31.09, G31.8, G31.89, G32.89, G93.8, G93.9, G94, G91.1, G31.9, G25.3, G95.19, G95.89, G90.9, Q85.1  G80  G40.311, G40.301, G40.211, G40.219, G40.411, G40.419, G40.111, G40.119, G40.804, G40.911, G40.919  G37.1, G37.2, G37.8, G81.90, G82.90, G82.50-G82.54, G83.5, G83.9, G93.1, G93.5, R40.3, 0016070, 0016071, 0016072, 0016073, 0016074, 0016075, 0016076, 0016077, 0016078, 001607B, 0016370, 0016371, 0016372, 0016373, 0016374, 0016375, 0016376, 0016377, 0016378, 001637B, 001U074, 001U076, 001U077, 001U079, 001U374, 001U376, 001U377, 001U379, 00B70ZZ, 00B73ZZ, 00B74ZZ, 00T70ZZ, 00T73ZZ, 00T74ZZ  I63.30, I63.50  G71, G72  G10, G20, G21.0, G21.11, G21.19, G21.8, G23.0-G23.2, G23.8, G24.02, G24.8, G25.3-G25.5, G25.81-G25.83, G25.89, G25.9, G80.3  T85.09XA, T85.190A, T85.192A, T85.199A, T85.79XA, Z98.2, Z45.41, Z45.42, 00160J0, 00160J1, 00160J2, 00160J3, 00160J4, 00160J5, 00160J6, 00160J7, 00160J8, 00160JB, 00160K0, 00160K1, 00160K2, 00160K3, 00160K4, 00160K5, 00160K6, 00160K7, 00160K8, 00160KB, 00163J0, 00163J1, 00163J2, 00163J3, 00163J4, 00163J5, 00163J6, 00163J7, 00163J8, 00163JB, 00163K0, 00163K1, 00163K2, 00163K3, 00163K4, 00163K5, 00163K6, 00163K7, 00163K8, 00163KB, 001U0J4, 001U0J6, 001U0J7, 001U0J9, 001U0K4, 001U0K6, 001U0K7, 001U0K9, 001U3J4, 001U3J6, 001U3J7, 001U3J9, 001U3K4, 001U3K6, 001U3K7, 001U3K9, 009600Z, 009630Z, 009640Z, 00H00MZ, 00H03MZ, 00H04MZ, 00H60MZ, 00H63MZ, 00H64MZ, 00HE0MZ, 00HE3MZ, 00HE4MZ, 00HU0MZ, 00HU3MZ, 00HU4MZ, 00HV0MZ, 00HV3MZ, 00HV4MZ, 00W60JZ, 00W63JZ, 00W64JZ, 00WU0JZ, 00WU3JZ, 00WU4JZ, 01HY0MZ, 01HY3MZ, 01HY4MZ, 0DH60MZ, 0DH63MZ, 0DH64MZ, 0W110J9, 0W110JB, 0W110JG, 0W110JJ, 3E1Q38X, 3E1Q38Z |
| Congenital or Genetic Defects | Q90.9, Q91.3, Q91.4, Q91.7, Q92.8, Q93, Q95.0, Q96.9, Q97, Q98, Q99.8, Q99.9  E34.3, M41.0, M41.2, M41.30, M41.8, M41.9, M43.30, M96.5, Q72.2, Q75.0, Q75.2, Q75.9, Q76.0-Q76.2, Q76.4-Q76.7, Q77, Q78.0-Q78.4, Q78.8, Q78.9  K44.9, Q79.0-Q79.5, Q79.9, Q79.59  Q81, Q87.1-Q87.3, Q87.40, Q87.81, Q87.89, Q89.7, Q89.9, Q99.2 |
| Renal and Urologic | Q60-Q64  N18  Z90.5, Z90.6, 0T160Z8, 0T160ZA, 0T164Z8, 0T164ZA, 0T170Z8, 0T170ZA, 0T174Z8, 0T174ZA, 0T180Z8, 0T180ZA, 0T184Z8, 0T184ZA, 0TB60ZZ, 0TB63ZZ, 0TB64ZZ, 0TB67ZZ, 0TB68ZZ, 0TB70ZZ, 0TB73ZZ, 0TB74ZZ, 0TB77ZZ, 0TB78ZZ, 0TT00ZZ, 0TT04ZZ, 0TT10ZZ, 0TT14ZZ, 0TT20ZZ, 0TT24ZZ, 0TT60ZZ, 0TT64ZZ, 0TT67ZZ, 0TT68ZZ, 0TT70ZZ, 0TT74ZZ, 0TT77ZZ, 0TT78ZZ, 0TTB0ZZ, 0TTB4ZZ, 0TTB7ZZ, 0TTB8ZZ, 0TTD0ZZ, 0TTD4ZZ, 0TTD7ZZ, 0TTD8ZZ  G83.4, N31.2, N31.9  T85.71XA, Z93.50-Z93.52, Z93.59, Z93.6, Z91.15, Z99.2, Z43.5, Z43.6, Z46.6, 031209D, 031209F, 03120AD, 03120AF, 03120JD, 03120JF, 03120KD, 03120KF, 03120ZD, 03120ZF, 031309D, 031309F, 03130AD, 03130AF, 03130JD, 03130JF, 03130KD, 03130KF, 03130ZD, 03130ZF, 031409D, 031409F, 03140AD, 03140AF, 03140JD, 03140JF, 03140KD, 03140KF, 03140ZD, 03140ZF, 031509D, 031509F, 03150AD, 03150AF, 03150JD, 03150JF, 03150KD, 03150KF, 03150ZD, 03150ZF, 031609D, 031609F, 03160AD, 03160AF, 03160JD, 03160JF, 03160KD, 03160KF, 03160ZD, 03160ZF, 031709D, 031709F, 03170AD, 03170AF, 03170JD, 03170JF, 03170KD, 03170KF, 03170ZD, 03170ZF, 031809D, 031809F, 03180AD, 03180AF, 03180JD, 03180JF, 03180KD, 03180KF, 03180ZD, 03180ZF, 031909F, 03190AF, 03190JF, 03190KF, 03190ZF, 031A09F, 031A0AF, 031A0JF, 031A0KF, 031A0ZF, 031B09F, 031B0AF, 031B0JF, 031B0KF, 031B0ZF, 031C09F, 031C0AF, 031C0JF, 031C0KF, 031C0ZF, 03WY0JZ, 03WY3JZ, 03WY4JZ, 03WYXJZ, 05HY33Z, 06HY33Z, 0JH60WZ, 0JH60XZ, 0JH63WZ, 0JH63XZ, 0JH80WZ, 0JH80XZ, 0JH83WZ, 0JH83XZ, 0JHD0WZ, 0JHD0XZ, 0JHD3WZ, 0JHD3XZ, 0JHF0WZ, 0JHF0XZ, 0JHF3WZ, 0JHF3XZ, 0JHL0WZ, 0JHL0XZ, 0JHL3WZ, 0JHL3XZ, 0JHM0WZ, 0JHM0XZ, 0JHM3WZ, 0JHM3XZ, 0T130ZB, 0T134ZB, 0T140ZB, 0T144ZB, 0T16079, 0T1607C, 0T1607D, 0T160J9, 0T160JC, 0T160JD, 0T160K9, 0T160KC, 0T160KD, 0T160Z9, 0T160ZC, 0T160ZD, 0T163JD, 0T16479, 0T1647C, 0T1647D, 0T164J9, 0T164JC, 0T164JD, 0T164K9, 0T164KC, 0T164KD, 0T164Z9, 0T164ZC, 0T164ZD, 0T17079, 0T1707C, 0T1707D, 0T170J9, 0T170JC, 0T170JD, 0T170K9, 0T170KC, 0T170KD, 0T170Z9, 0T170ZC, 0T170ZD, 0T173JD, 0T17479, 0T1747C, 0T1747D, 0T174J9, 0T174JC, 0T174JD, 0T174K9, 0T174KC, 0T174KD, 0T174Z9, 0T174ZC, 0T174ZD, 0T18079, 0T1807C, 0T1807D, 0T180J9, 0T180JC, 0T180JD, 0T180K9, 0T180KC, 0T180KD, 0T180Z9, 0T180ZC, 0T180ZD, 0T183JD, 0T18479, 0T1847C, 0T1847D, 0T184J9, 0T184JC, 0T184JD, 0T184K9, 0T184KC, 0T184KD, 0T184Z9, 0T184ZC, 0T184ZD, 0T1B0ZD, 0T1B4ZD, 0T25X0Z, 0T29X0Z, 0T29XYZ, 0T2BX0Z, 0T9000Z, 0T9030Z, 0T9040Z, 0T9070Z, 0T9080Z, 0T9100Z, 0T9130Z, 0T9140Z, 0T9170Z, 0T9180Z, 0T9370Z, 0T9380Z, 0T9470Z, 0T9480Z, 0TQ67ZZ, 0TQ77ZZ, 3E1K38Z, 3E1M39Z, 5A1D60Z  T86.10-T86.12, Z94.0, 0TY00Z0, 0TY00Z1, 0TY00Z2, 0TY10Z0, 0TY10Z1, 0TY10Z2 |
| Respiratory | Q30-Q34, P280  G47.35, I27.82, I43, J84.112, J96.20, Z90.2  E84  0B110Z4, 0B113Z4, 0B114Z4, 0BTC0ZZ, 0BTC4ZZ, 0BTD0ZZ, 0BTD4ZZ, 0BTF0ZZ, 0BTF4ZZ, 0BTG0ZZ, 0BTG4ZZ, 0BTJ0ZZ, 0BTJ4ZZ, 0BTK0ZZ, 0BTK4ZZ, 0BTL0ZZ, 0BTL4ZZ, 0BTM0ZZ, 0BTM4ZZ, 0CTS0ZZ, 0CTS4ZZ, 0CTS7ZZ, 0CTS8ZZ  J95.00-J95.04, J95.09, Z43.0, Z93.0, Z99.0, J95.850, Z99.11, Z99.12, 0B110F4, 0B113F4, 0B114F4, 0B21XFZ, 0BHR0MZ, 0BHR3MZ, 0BHR4MZ, 0BHS0MZ, 0BHS3MZ, 0BHS4MZ, 0BW10FZ, 0BW13FZ, 0BW14FZ, 0JH604Z, 0JH634Z, 0JH804Z, 0JH834Z, 0WQ6XZ2, 3E1F78Z  T86.810, T86.811, T86.819, Z94.2, 0BYC0Z0, 0BYC0Z1, 0BYC0Z2, 0BYD0Z0, 0BYD0Z1, 0BYD0Z2, 0BYF0Z0, 0BYF0Z1, 0BYF0Z2, 0BYG0Z0, 0BYG0Z1, 0BYG0Z2, 0BYH0Z0, 0BYH0Z1, 0BYH0Z2, 0BYJ0Z0, 0BYJ0Z1, 0BYJ0Z2, 0BYK0Z0, 0BYK0Z1, 0BYK0Z2, 0BYL0Z0, 0BYL0Z1, 0BYL0Z2, 0BYM0Z0, 0BYM0Z1, 0BYM0Z2 |

All codes are taken from Feudtner, C., Feinstein, J.A., Zhong, W., Hall, M. & Dai, D. Pediatric complex chronic conditions classification system version 2: updated for ICD-10 and complex medical technology dependence and transplantation. BMC Pediatr 14, 199 (2014).

**Supplementary Figure 2: Sex-Stratified Propensity Score Density Before and After Matching**


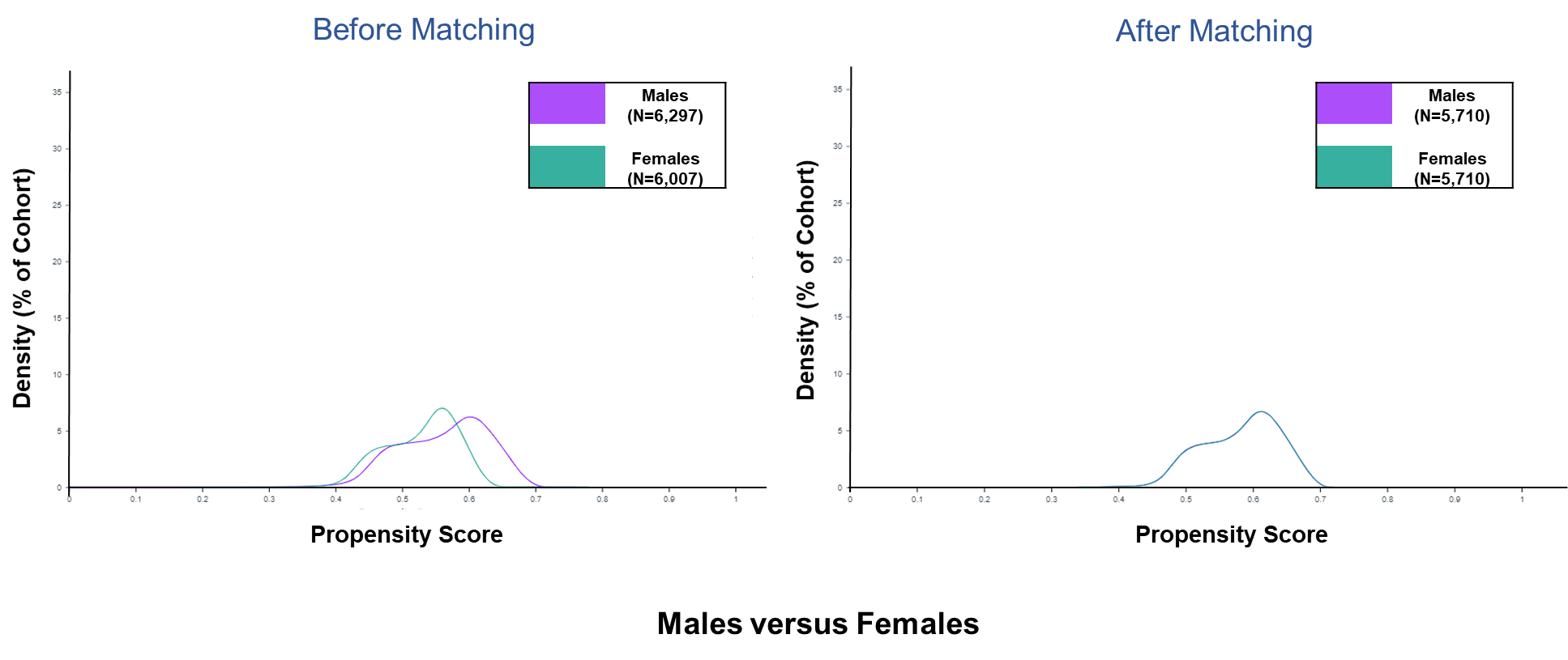


**Supplementary Figure 3: Race-Stratified Propensity Score Density Before and After Matching: Non-Hispanic Blacks versus Non-Hispanic Whites**

**
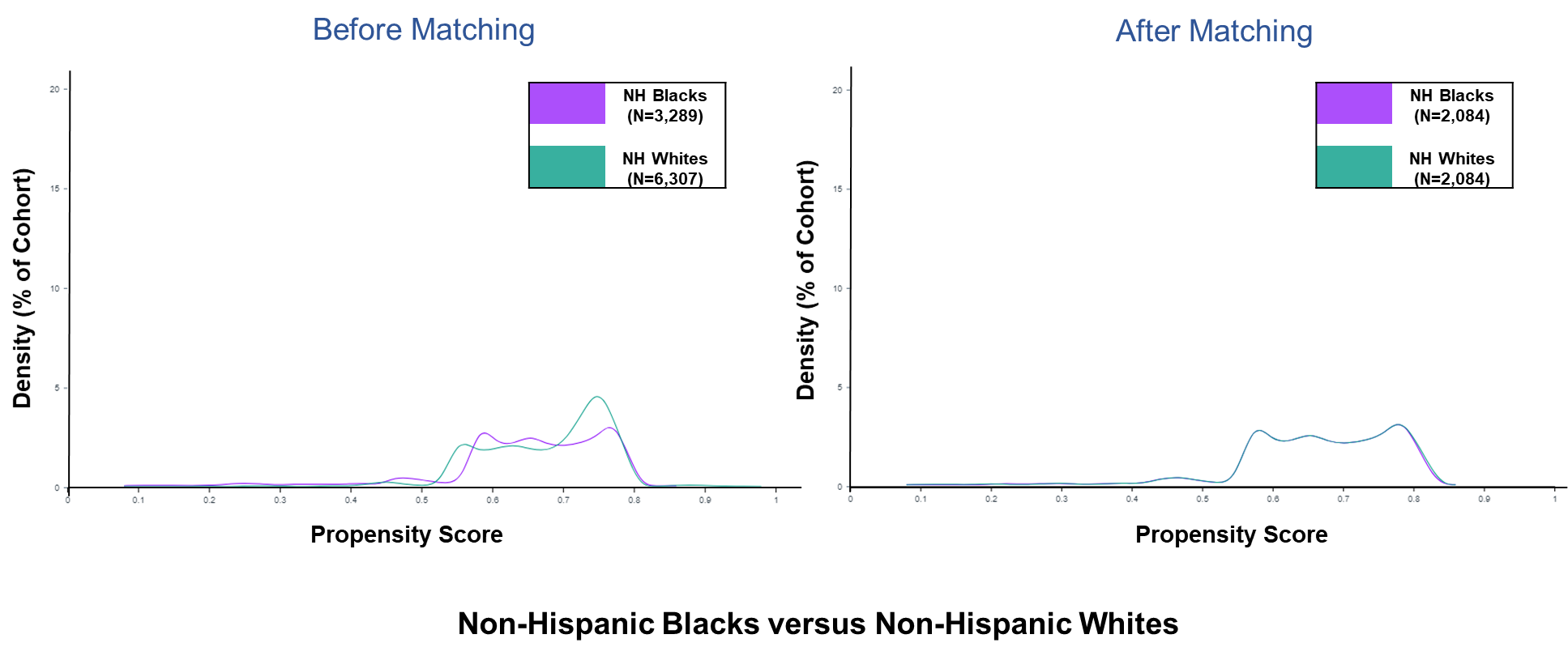
**

**Supplementary Figure 4: Race-Stratified Propensity Score Density Before and After Matching: Hispanics versus Non-Hispanic Whites**


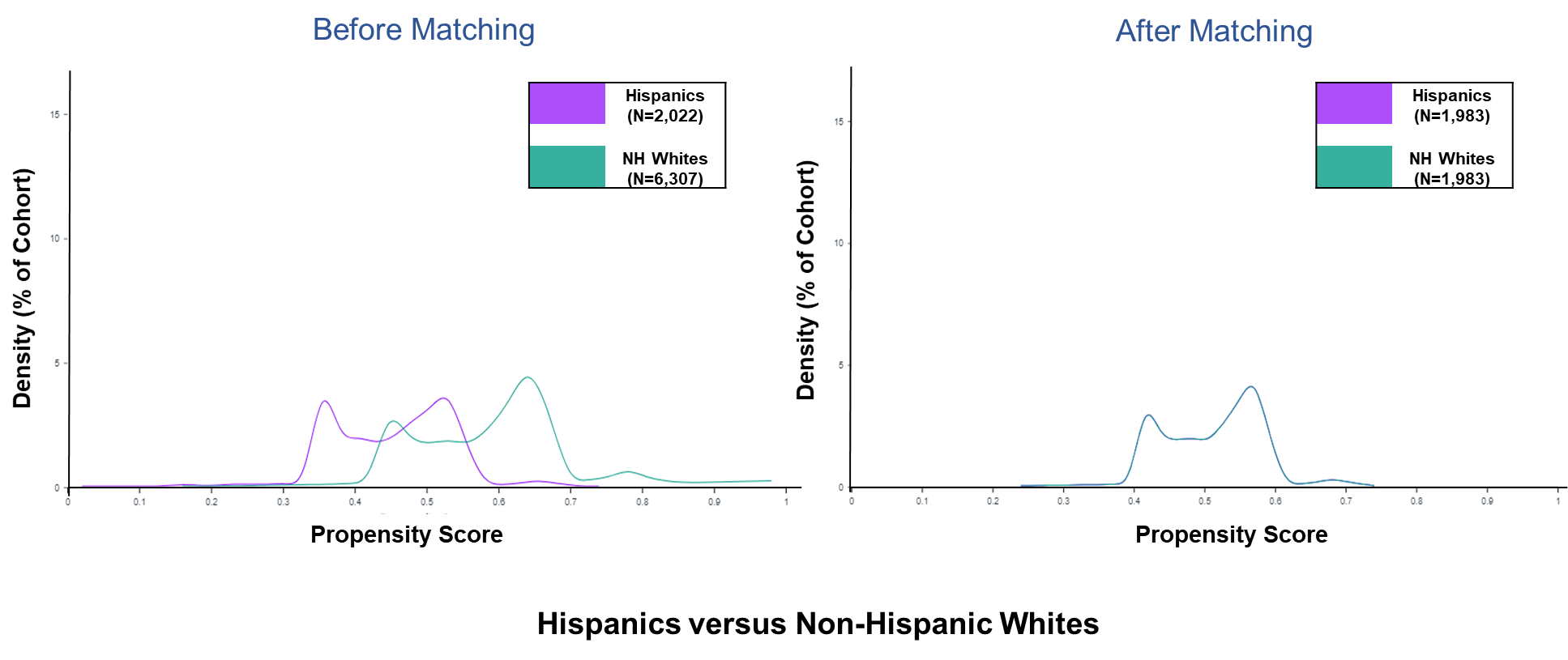


**
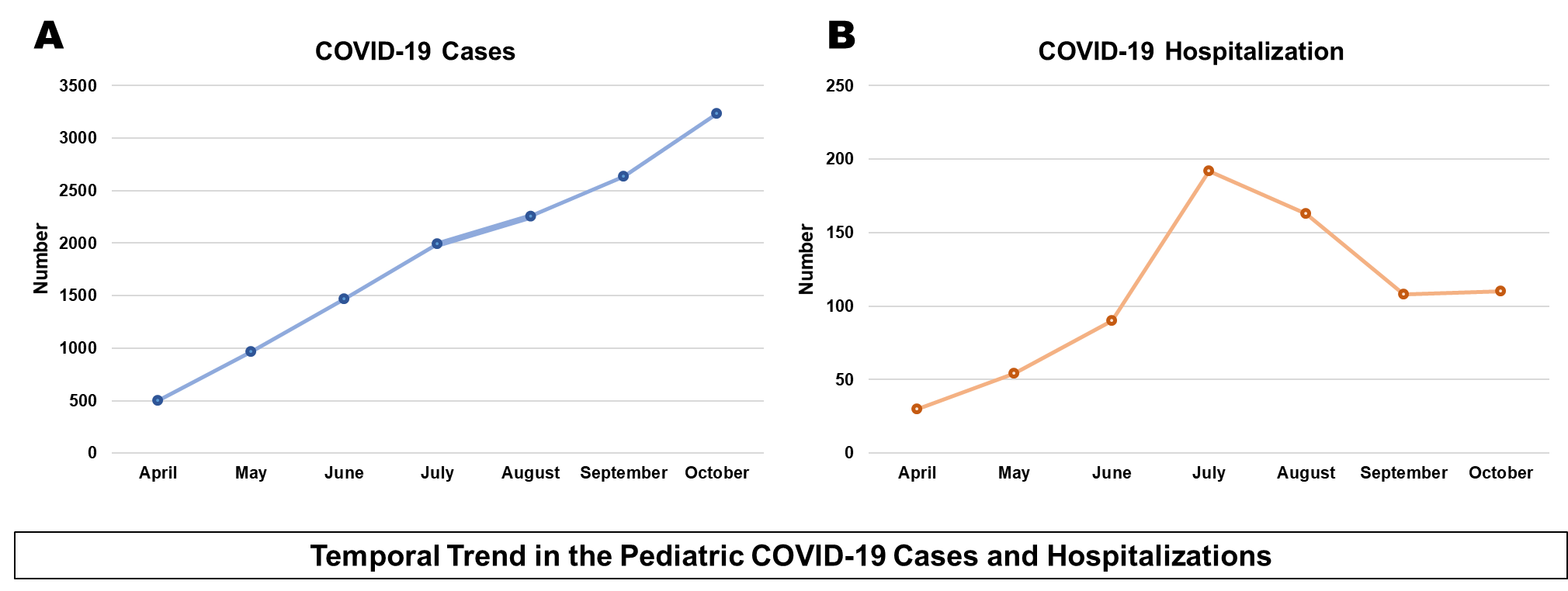
Supplementary Figure 5: Temporal Trend in Pediatric COVID-19 Cases and Hospitalization**

**References**

1 National Center for Advancing Translational Sciences. National COVID Cohort Collaborative (N3C). Available at: <https://covid.cd2h.org/N3C>. Accessed on: October 30, 2020.

2 Food & Drugs Administration. FDA Reporter TriNetX. Available at: <https://fdareporter.com/organizations/646531868-trinetx>. Accessed on: October 30th, 2020.

3 Cision PR Newswire. TriNetX Signs Agreement with FDA Sentinel Program. Available at: <https://www.prnewswire.com/news-releases/trinetx-signs-agreement-with-fda-sentinel-program-301092609.html>. Accessed on October 30th, 2020.

4 Centers for Disease Control and Prevention. School Decision-Making Tool for Parents, Caregivers, and Guardians. Available at: <https://www.cdc.gov/coronavirus/2019-ncov/community/schools-childcare/decision-tool.html>. Accessed on October 30th, 2020.

5 Harrison, S. L., Fazio-Eynullayeva, E., Lane, D. A., Underhill, P. & Lip, G. Y. H. Comorbidities associated with mortality in 31,461 adults with COVID-19 in the United States: A federated electronic medical record analysis. *PLoS Med* **17**, e1003321, doi:10.1371/journal.pmed.1003321 (2020).

6 Singh, S. *et al.* Risk of Severe Coronavirus Disease 2019 in Patients With Inflammatory Bowel Disease in the United States: A Multicenter Research Network Study. *Gastroenterology*, doi:10.1053/j.gastro.2020.06.003 (2020).

7 Singh, S. & Khan, A. Clinical Characteristics and Outcomes of Coronavirus Disease 2019 Among Patients With Preexisting Liver Disease in the United States: A Multicenter Research Network Study. *Gastroenterology* **159**, 768-771 e763, doi:10.1053/j.gastro.2020.04.064 (2020).

8 Alkhouli, M., Nanjundappa, A., Annie, F., Bates, M. C. & Bhatt, D. L. Sex Differences in Case Fatality Rate of COVID-19: Insights From a Multinational Registry. *Mayo Clin Proc* **95**, 1613-1620, doi:10.1016/j.mayocp.2020.05.014 (2020).

9 Taquet, M., Geddes, J. R., Husain, M., Luciano, S. & Harrison, P. J. 6-month neurological and psychiatric outcomes in 236 379 survivors of COVID-19: a retrospective cohort study using electronic health records. *The Lancet Psychiatry*, doi:10.1016/S2215-0366(21)00084-5.
